# Changes in gut microbiota before and after treatment in patients with primary hypothyroidism

**DOI:** 10.1515/biol-2025-1307

**Published:** 2026-04-17

**Authors:** Qian Xu, JiaSheng Ju, Xiang Han, YaPing Gu, JiaYun Li, HaiBing Ju

**Affiliations:** Department of Endocrinology, 920th Hospital of the Joint Logistics Support Force of the Chinese People’s Liberation Army, Kunming, China; Department of Endocrinology, Bijie Traditional Chinese Medicine Hospital, Bijie, China; Department of Neurosurgery, Zhujiang Hospital of Southern Medical University, Guangzhou, China

**Keywords:** primary hypothyroidism, Hashimoto’s thyroiditis, gut microbiota, thyroid-gut axis, levothyroxine sodium

## Abstract

This study aims to analyze the differences in gut microbiota of patients with primary hypothyroidism before and after treatment and their correlations with clinical indicators. A total of 20 newly diagnosed primary hypothyroidism patients were selected for the study. These patients were treated with levothyroxine sodium (L-T4) for 12 weeks, and fecal samples were collected before and after treatment. Concurrently, fecal samples from 20 healthy controls were also collected. High-throughput 16sRNA sequencing was conducted to analyze the composition of gut microbiota in each group. At the phylum level, the abundance of Bacteroidetes decreased in patients with primary hypothyroidism. After treatment, the abundance of Bacteroidetes increased, while the abundance of Proteobacteria and Desulfobacterota decreased. At the genus level, the abundance of *Bacteroides* and *Faecalibacterium* decreased, while the abundance of *Collinsella*, *Klebsiella*, *Parabacteroides*, and the genus *Koalarothia* increased. After treatment, the abundance of *Streptococcus*, *Ruminococcusgnavus*, *Megamonas* and *Corrococcus* increased, while the abundance of *Escherichia-Shigella*, *Agaricus*, *Ruminococcus torques*, *Koalarothia*, and *Blautia* decreased. Differential species screening showed 24 different species between the pre-treatment and post-treatment groups. Spearman correlation analysis showed that *Streptococcus* was positively correlated with TT3, TT4, and FT4, and negatively correlated with AST; *R. torques* was negatively correlated with TT4 and FT4; Koalarothia was positively correlated with TgAb and TC; *Blautia* was positively correlated with TPOAb. Patients with primary hypothyroidism exhibit gut microbiota dysbiosis, with a decrease in the abundance of certain short-chain fatty acid-producing bacteria, which increases after treatment. There is a certain correlation between specific gut microbiota and thyroid function as well as lipid metabolism indicators.

## 1 Introduction

Primary hypothyroidism is a disease characterized by a deficiency of thyroid hormones due to intrinsic thyroid gland pathology such as autoimmunity, post-radioiodine therapy for hyperthyroidism, or thyroid surgery. Diagnosis primarily relies on the assessment of thyroid function, and the clinical presentation may vary by age and gender. In severe cases, it can lead to cardiovascular, neurological, cognitive, and metabolic dysfunction, significantly impacting patients’ quality of life [1]. The mainstay of treatment is levothyroxine replacement therapy. Hashimoto’s thyroiditis (HT), a form of autoimmune thyroiditis (AITD), also known as chronic lymphocytic thyroiditis, is the leading cause of primary hypothyroidism.

In recent years, the concept of the thyroid-gut axis (TGA) has been proposed [2], which suggests that the thyroid, originating from endodermal cells, may be influenced by gastrointestinal microbiota that affect thyroid hormone metabolism and function. Research indicates that gut microbiota dysbiosis can influence the occurrence and development of hypothyroidism through mechanisms such as mediating immune dysregulation, increasing intestinal permeability, regulating chronic inflammation, and causing malabsorption of nutrients [3]. Taking key trace elements as an example, zinc, a necessary substance for the synthesis of thyroid hormones, is absorbed in the stomach, duodenum, and jejunum. Gut microbiota can affect the synthesis of thyroid hormones by influencing zinc absorption [4]. The active center of thyroid peroxidase contains zinc, which can enhance the activity of type II iodothyronine deiodinase (DIO2), a key enzyme that promotes the conversion of T4 to active T3. Hence, zinc deficiency could contribute to the development of hypothyroidism [5]. Notably, primary hypothyroidism itself can reduce the integrity of the gastric mucosa, decrease gastric acid secretion, alter the mucosal environment, and disrupt the composition of the gut microbiota, thereby affecting the absorption of trace elements and nutrients. Thus, there exists a complex bidirectional regulatory relationship between hypothyroidism and gut microbiota dysbiosis [6]. This study employed 16sRNA gene sequencing technology to analyze the differences in gut microbiota composition between healthy controls and newly diagnosed patients with Hashimoto’s thyroiditis accompanied by primary hypothyroidism before and after treatment, aiming to explore the changes in gut microbiota and their correlation with clinical indicators before and after treatment in these patients.

## 2 Subjects and methods

### 2.1 Subjects

The study enrolled 48 newly diagnosed patients with Hashimoto’s thyroiditis accompanied by primary hypothyroidism who visited the hospital between May 2022 and May 2023. After excluding 28 patients who were lost to follow-up or did not adhere to medication, 20 patients were included in the analysis (7 males and 13 females, aged 55.55 ± 12.87 years). All patients received levothyroxine sodium (Germany’s Merck) replacement therapy, with an initial dose of 25–50 μg/d, adjusted according to TSH levels until the required dose was achieved. A healthy control group of 20 individuals (7 males and 13 females, aged 53.82 ± 13.36 years) was also enrolled.

#### 2.1.1 Inclusion criteria

Inclusion criteria for patients with primary hypothyroidism: all selected patients met the diagnostic criteria for Hashimoto’s thyroiditis and exhibited clinical hypothyroidism; age >18 years, of Han ethnicity, and long-term residents of Kunming and surrounding areas; regularly received L-T4 replacement therapy during outpatient or inpatient care.

Inclusion criteria for healthy control group: (1) all individuals were of Han ethnicity and long-term residents of Kunming and surrounding areas; (2) thyroid ultrasound did not indicate thyroid abnormalities, thyroid function was normal, and thyroid-related antibodies were within the normal range; (3) no significant abnormalities were detected in abdominal ultrasound, liver and kidney function, blood routine, and urinalysis.

#### 2.1.2 Exclusion criteria

(1) History of thyroid disease;
(2) Pregnant, smokers, alcohol abusers, patients with diabetes, liver or kidney dysfunction, diarrhea, or gastritis;
(3) Recent use of antibiotics, probiotics, hormones, laxatives, proton pump inhibitors, insulin sensitizers, amiodarone, or traditional Chinese medicine within the past 3 months;
(4) Known autoimmune diseases, acute or chronic illnesses, brain-related diseases (such as Parkinson’s or dementia), malignant tumors, or a history of any gastrointestinal surgery.

**Informed consent:** Informed consent has been obtained from all individuals included in this study.

**Ethical approval:** The research related to human use has been complied with all the relevant national regulations, institutional policies and in accordance with the tenets of the Helsinki Declaration, and has been approved by the 920th Hospital of the Joint Logistics Support Force of the Chinese People’s Liberation Army Ethics Committee (Ethics Review 2022-128-01).

### 2.2 Clinical data and sample collection

Patient gender, age, height, weight, liver function, and thyroid function, as well as relevant antibody detection indicators, were collected. The body mass index (BMI) was calculated as weight/height squared (kg/m^2^). Blood samples were collected before and after treatment, with fasting for over 8 h required before sample collection. Blood was drawn the following morning on an empty stomach for testing thyroid function, lipid profile, and liver function in the hospital’s laboratory department. Stool samples were collected from both the healthy control group and the patients before and 12 weeks after treatment. These samples were immediately sealed, labeled, and stored in a −80 °C freezer for further analysis.

### 2.3 Experimental procedures

#### 2.3.1 DNA extraction, amplification, and purification of stool samples

Total microbial DNA was extracted from all samples using cetyltrimethylammonium bromide (CTAB) as a detergent. The quality and integrity of the extracted DNA were assessed using spectrophotometry and electrophoresis. The extracted metagenomic DNA served as a template for multi-primer amplification via multiple PCR and high-fidelity, low-cycle PCR systems. Amplification products were purified using the Am pure XT beads (Agencourt) reagent kit.

#### 2.3.2 Sequencing and data analysis

The library was evaluated using the Agilent 2100 Bioanalyzer and the Illumina reagent kit, with libraries having a concentration above 2 nM considered qualified. Dual-end sequencing (2 × 250 bp) was performed using the NovaSeq 6000 sequencer. According to the standard operating procedures of the Illumina platform, qualified sequences were sequenced to obtain raw data, followed by quality control and chimera filtering to obtain high-quality target region sequences. An OTU-like table was constructed using the ASVs concept, yielding the final ASV feature table and feature sequences. Further analyses, including diversity analysis, species classification annotation, and differential analysis, were performed.

### 2.4 Statistical analysis

Statistical analysis and visualization were conducted using R4.2.1, Origin2021b, and SPSS 29.0. SPSS 29.0 was used for statistical analysis. Data from normally distributed variables were described as mean ± standard deviation (SD), while non-normally distributed variables were presented as median (interquartile range, IQR). For comparison of means between two groups, a two-sample *t*-test or Wilcoxon rank-sum test was used depending on whether the data were normally distributed. For comparisons between three groups, one-way ANOVA or the Kruskal-Wallis test was employed. The correlation analysis used Spearman’s correlation coefficient (*ρ*). The threshold for statistical significance was set at *p* < 0.05.

## 3 Results

### 3.1 Analysis of clinical indicators before and after treatment in patients with primary hypothyroidism

After treatment, patients with primary hypothyroidism showed a statistically significant increase in TT3, TT4, FT3, and FT4 (*p* < 0.01). In contrast, there were statistically significant decreases in WG, BMI, TSH, TgAb, TPOAb, AST, TG, TC, and LDL (*p* < 0.05), as shown in Table 1.

**Table 1:** Comparison of clinical indicators before and after treatment in patients with newly diagnosed primary hypothyroidism.

Variable	Before treatment	After treatment	*t*/*Z*	*p*
WG (kg)	65.85 ± 12.474	65.20 ± 11.710	2.292	0.017
BMI (kg/m^2^)	24.864 ± 3.975	24.632 ± 3.755	2.205	0.020
TT_3_ (ng/ml)	0.91(0.68, 1.22)	1.63(1.38, 1.89)	−3.771	<0.01
TT_4_ (μg/dl)	39.65(14.07, 52.37)	102.35(77.07, 136.40)	−3.920	<0.001
TSH (μIU/ml)	55.96(22.27, 123.86)	4.27(3.95, 4.67)	−3.920	<0.001
FT_3_ (pmol/l)	3.05(2.33, 3.79)	4.63(4.03, 5.07)	−3.547	<0.001
FT_4_ (pmol/l)	8.22(4.60, 9.79)	16.291(14.21, 18.56)	−3.920	<0.001
TgAb (IU/ml)	52.795(29.220, 68.012)	35.505(20.295, 53.992)	−2.949	0.003
TPOAb (U/ml)	372.23(83.33, 1819.84)	112.66(40.41, 536.13)	−3.696	<0.001
ALB (g/l)	37.75(35.87, 42.35)	38.89(37.27, 44.25)	−1.120	0.263
ALT (U/l)	21.50 (15.50, 38.50)	19.50(16.00, 27.50)	−0.946	0.344
AST (U/l)	21.00(19.25, 29.50)	16.50(15.00, 21.75)	−2.741	0.006
TG (mmol/l)	1.90(1.13, 2.64)	1.41(0.95, 1.91)	−3.211	0.001
TC (mmol/l)	5.76(3.84, 6.87)	4.62(3.81, 5.28)	−2.203	0.028
HDL (mmol/l)	0.97(0.79, 1.50)	1.27(0.95, 1.46)	−1.120	0.263
LDL (mmol/l)	3.09(2.44, 4.49)	3.05(1.92, 3.67)	−2.688	0.007

### 3.2 Analysis of gut microbiota diversity

**Figure 1:**
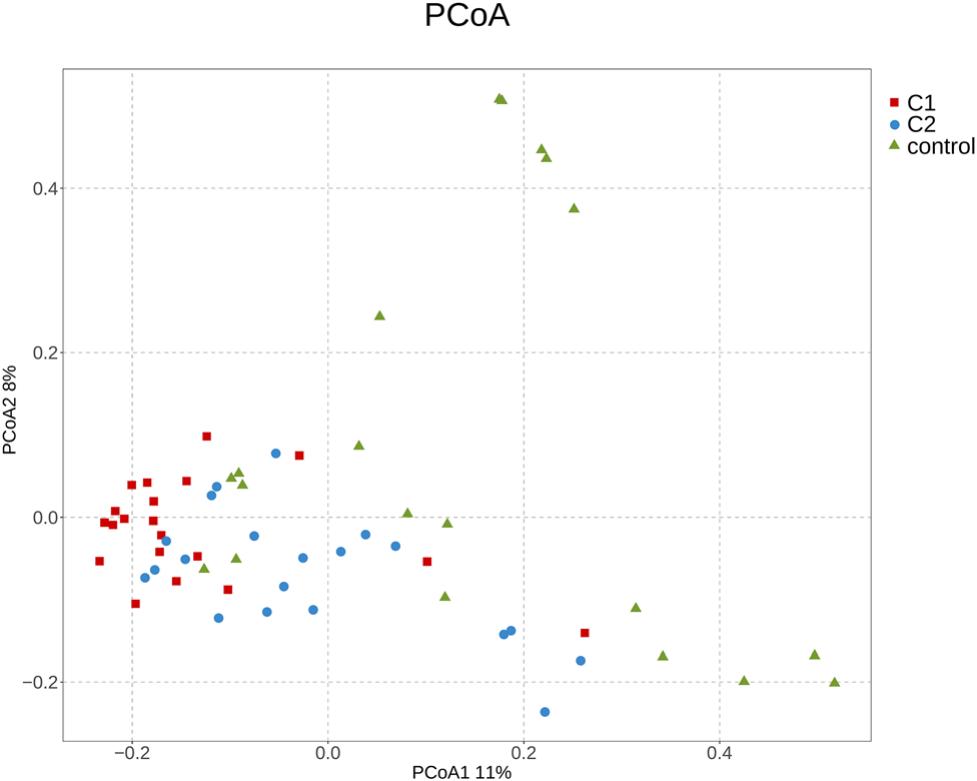
Principal coordinate analysis (PCoA) of gut microbiota. This plot illustrates the structural differences of gut microbial communities among the three groups: C1: HT with hypothyroidism pre-treatment group; C2: HT with hypothyroidism post-treatment group; control: healthy controls.

Alpha diversity, based on species richness and evenness, reflects species diversity within a specific region or ecosystem. The Chao1 index indicated a statistically significant difference between the pre-treatment group and the healthy control group (*p* < 0.05). However, no statistically significant differences were observed among the three groups for the Pielou index, Shannon index, or Simpson index, as shown in Table 2. Beta diversity, which represents species differences between environments, was assessed through principal coordinate analysis (PCoA), revealing structural differences among the three groups, as depicted in Figure 1.

**Table 2:** Alpha diversity index comparison of each group (mean ± SD).

α-Diversity	Healthy controls	Before treatment	After treatment	*t* ^1^	*t* ^2^	*p* ^1^	*p* ^2^
Chao1	289.0 ± 69.357	343.82 ± 76.995	316.414 ± 69.064	2.365	1.767	0.023	0.093
Pielou_e	0.664 ± 0.068	0.668 ± 0.068	0.679 ± 0.061	0.182	−0.438	0.856	0.666
Shannon	5.411 ± 0.697	5.623 ± 0.746	5.630 ± 0.639	0.932	−0.027	0.357	0.979
Simpson	0.928 ± 0.044	0.941 ± 0.030	0.944 ± 0.026	1.044	−0.287	0.152	0.777

*t* ^1^: *t* value of newly diagnosed hypothyroidism patients before treatment compared with the control group; *t* ^2^: *t* value of hypothyroidism patients before and after treatment; *p* ^1^: *p* value of newly diagnosed hypothyroidism patients before treatment compared with the control group; *p* ^2^: comparison of *p* values before and after treatment in patients with hypothyroidism.

### 3.3 Comparison of gut microbiota relative abundance at the phylum level

**Figure 2:**
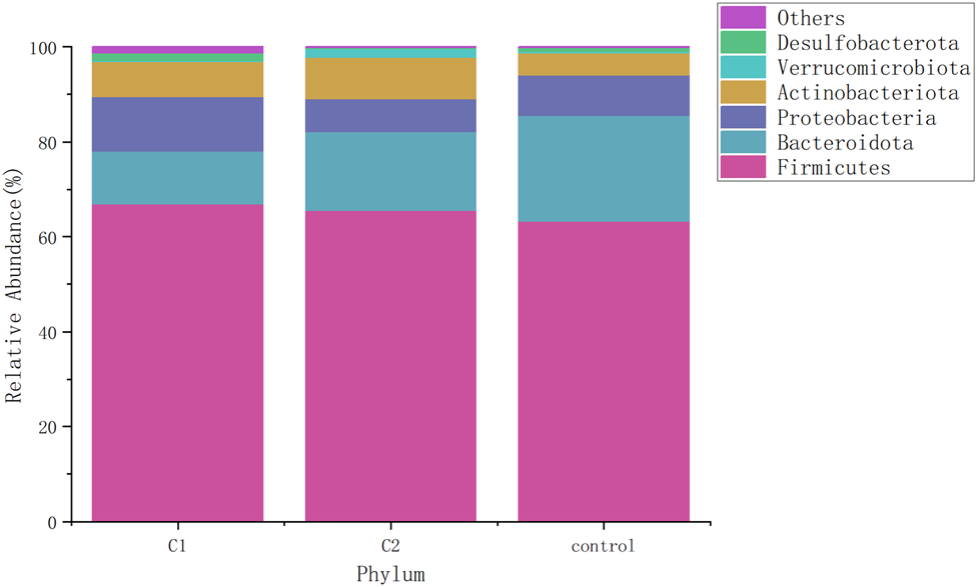
Bar graph of relative abundance of gut microbiota at phylum level. The results of the gut microbiome at the phylum level were displayed by the percentage columnar stack plot, which showed that the intestinal flora in the three groups was dominated by Firmicutes, Bacteroidetes, Proteobacteria, and Actinobacteria. C1: HT with hypothyroidism pre-treatment group; C2: HT with hypothyroidism post-treatment group; control: healthy controls.

Figure 2 illustrates the relative abundance of gut microbiota at the phylum level across the three groups. The gut microbiota in all three groups primarily consisted of Firmicutes, Bacteroidetes, Proteobacteria, and Actinobacteria. Compared to the healthy control group, the newly diagnosed hypothyroidism patients exhibited a decrease in Bacteroidetes abundance (*p* < 0.05). Post-treatment, there was a significant increase in Bacteroidetes abundance and a decrease in Proteobacteria and Desulfobacterota abundances (p < 0.05), as shown in Table 3.

**Table 3:** Comparison of gut microbiota relative abundance at the phylum level in each group.

Phylum	Healthy controls	Before treatment	After treatment	*t* ^1^/*Z* ^1^	*p* ^1^	*t* ^2^/*Z* ^2^	*p* ^2^
Firmicutes	63.332 ± 11.631	64.158 ± 10.738	68.538 ± 10.519	0.173	0.864	−1.459	0.165
Bacteroidota	22.328 ± 7.201	10.808 ± 4.277	17.369 ± 4.353	−5.006	0.001	−3.818	0.002
Proteobacteria	3.381(0.788, 13.233)	4.698(1.761, 24.191)	2.577(0.993, 14.252)	−0.859	0.417	−1.968	0.049
Actinobacteriota	4.363(1.198, 8.362)	5.510(2.477, 8.941)	4.869(1.112, 17.831)	−0.736	0.490	−0.621	0.534
Desulfobacterota	0.624(0.201, 1.286)	1.172(0.257, 2.855)	0.243(0.173, 0.326)	−1.227	0.238	−3.521	<0.001
*F*/*B*	2.571(1.769, 6.732)	6.991(4.164, 7.412)	3.922(2.997, 5.384)	−1.840	0.070	−2.796	0.005

*t* ^1^/*Z* ^1^: *t* value or *Z* value of newly diagnosed hypothyroidism patients before treatment compared with control group; *t* ^2^/*Z* ^2^: *t* value or *Z* value of patients with hypothyroidism before and after treatment; *p* ^1^: *p* value of newly diagnosed hypothyroidism patients before treatment compared with the control group; *p* ^2^: *p* values before and after treatment in patients with hypothyroidism; *F*/*B*: Firmicutes/Bacteroidetes.

### 3.4 Comparison of gut microbiota relative abundance at the genus level

**Figure 3:**
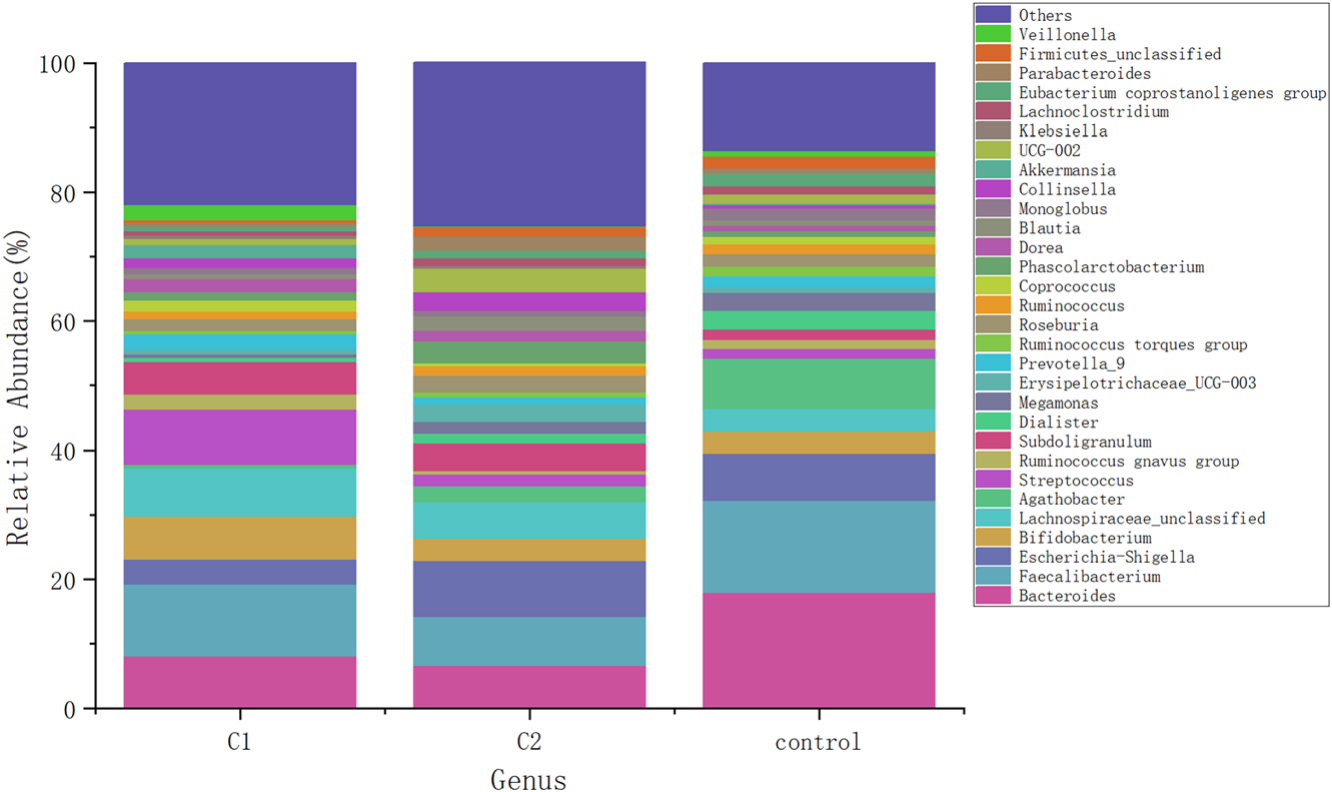
Bar graph of relative abundance of gut microbiota at genus level. The genus level abundance TOP30 species classification was selected, and the relative abundance of each group was presented in different forms. C1: HT with hypothyroidism pre-treatment group; C2: HT with hypothyroidism post-treatment group; control: healthy controls.

Figure 3 illustrates the relative abundance of gut microbiota at the genus level across the three groups. At the genus level, compared to the healthy control group, newly diagnosed hypothyroidism patients had significantly lower abundances of *Bacteroides* and *Faecalibacterium*, while the abundances of *Enterobacter*, *Collinsella*, *Klebsiella*, and *Parabacteroides* were significantly higher (*p* < 0.05). Post-treatment, there was a significant increase in *Streptococcus*, *Ruminococcus gnavus* group, *Megamonas* and *Coprococcus* abundances, while the abundances of *Escherichia-Shigella*, *Agarivorans*, *Ruminococcus torques* group, *Coriobacteriaceae*, and *Blautia* decreased (*p* < 0.05), as shown in Table 4.

**Table 4:** Comparison of gut microbiota relative abundance at the genus level in each group.

Genus	Healthy controls	Before treatment	After treatment	*Z* ^1^	*p* ^1^	*Z* ^2^	*p* ^2^
*Bacteroides*	13.791(4.961, 29.465)	5.039(4.407, 7.285)	6.902(3.051, 15.127)	−2.265	0.024	−0.466	0.641
*Faecalibacterium*	13.070(10.709, 20.435)	6.861(1.534, 13.119)	8.963(4.438, 22.277)	−2.567	0.010	−1.243	0.214
*Escherichia-Shigella*	0.719(0.513, 11.708)	1.287(0.731, 22.776)	0.763(0.508, 1.969)	−1.510	0.131	−1.968	0.049
*Bifidobacterium*	2.525(0.220, 7.245)	1.937(0.358, 7.257)	3.580(0.162, 11.646)	−0.604	0.546	−1.450	0.147
*Lachnospiraceae_unclassified*	2.281(1.249, 6.395)	4.823(0.791, 9.721)	5.308(2.198, 13.832)	−0.755	0.450	−1.191	0.234
*Agathobacter*	1.561(0.303, 15.003)	1.536(0.304, 5.556)	0.163(0.065, 1.509)	−1.208	0.227	−2.610	0.009
*Streptococcus*	0.485(0.457, 1.508)	1.187(0.223, 2.503)	4.949(3.091, 14.210)	−0.604	0.546	−2.537	0.011
*Ruminococcus gnavus* group	0.229(0.112, 3.273)	0.395(0.168, 1.039)	1.325(0.922, 4.427)	−0.755	0.450	−2.951	0.003
*Dialister*	0.719(0.208, 6.952)	0.296(0.032, 3.577)	0.100(0.050, 1.122)	−1.812	0.070	−1.191	0.234
*Megamonas*	0.128(0.445, 5.769)	0.074(0.018, 0.143)	0.383(0.002, 0.877)	−1.208	0.227	−2.123	0.034
*Ruminococcus torques* group	0.823(1.722, 3.264)	0.505(0.368, 1.425)	0.384(0.071, 1.211)	−0.302	0.763	−2.537	0.011
*Ruminococcus*	0.916(0.293, 1.739)	0.357(0.103, 3.406)	0.815(0.009, 2.497)	−0.151	0.880	−0.932	0.351
*Coprococcus*	0.741(0.184, 1.950)	0.269(0.038, 0.774)	0.673(0.045, 4.419)	−1.812	0.070	−2.175	0.030
*Phascolarctobacterium*	0.039(0.109, 2.306)	1.747(0.231, 4.329)	1.099(0.030, 2.848)	−2.416	0.016	−2.744	0.006
*Blautia*	0.640(0.304, 1.221)	1.041(0.681, 2.193)	0.829(0.526, 1.099)	−1.963	0.050	−2.382	0.017
*Collinsella*	0.137(0.025, 1.012)	1.276(1.045, 4.299)	0.138(0.133, 2.821)	−2.869	0.004	−1.139	0.255
*Klebsiella*	0.043(0.000, 0.056)	0.162(0.529, 0.865)	0.105(0.003, 0.321)	−2.758	0.006	−1.398	0.162
*Parabacteroides*	0.433(0.155, 1.145)	1.704(0.386, 4.024)	0.390(0.072, 1.037)	−2.265	0.024	−1.657	0.098
*Firmicutes*	0.413(0.144, 1.119)	0.379(0.245, 2.922)	0.486(0.255, 0.606)	−0.604	0.546	−1.036	0.300
*Veillonella*	0.082(0.032, 0.270)	0.054(0.010, 0.153)	0.101(0.042, 2.984)	−0.982	0.326	−1.398	0.162

*Z* ^1^: the *Z* value of newly diagnosed hypothyroidism patients before treatment was compared with that of the control group; *Z* ^2^: the *Z* scores of patients with hypothyroidism before and after treatment were compared; *p* ^1^: *p* values were compared between patients with newly diagnosed hypothyroidism before treatment and controls; *p* ^2^: comparison of *p* values before and after treatment in patients with hypothyroidism.

### 3.5 Selection of differential species among the three groups

**Figure 4:**
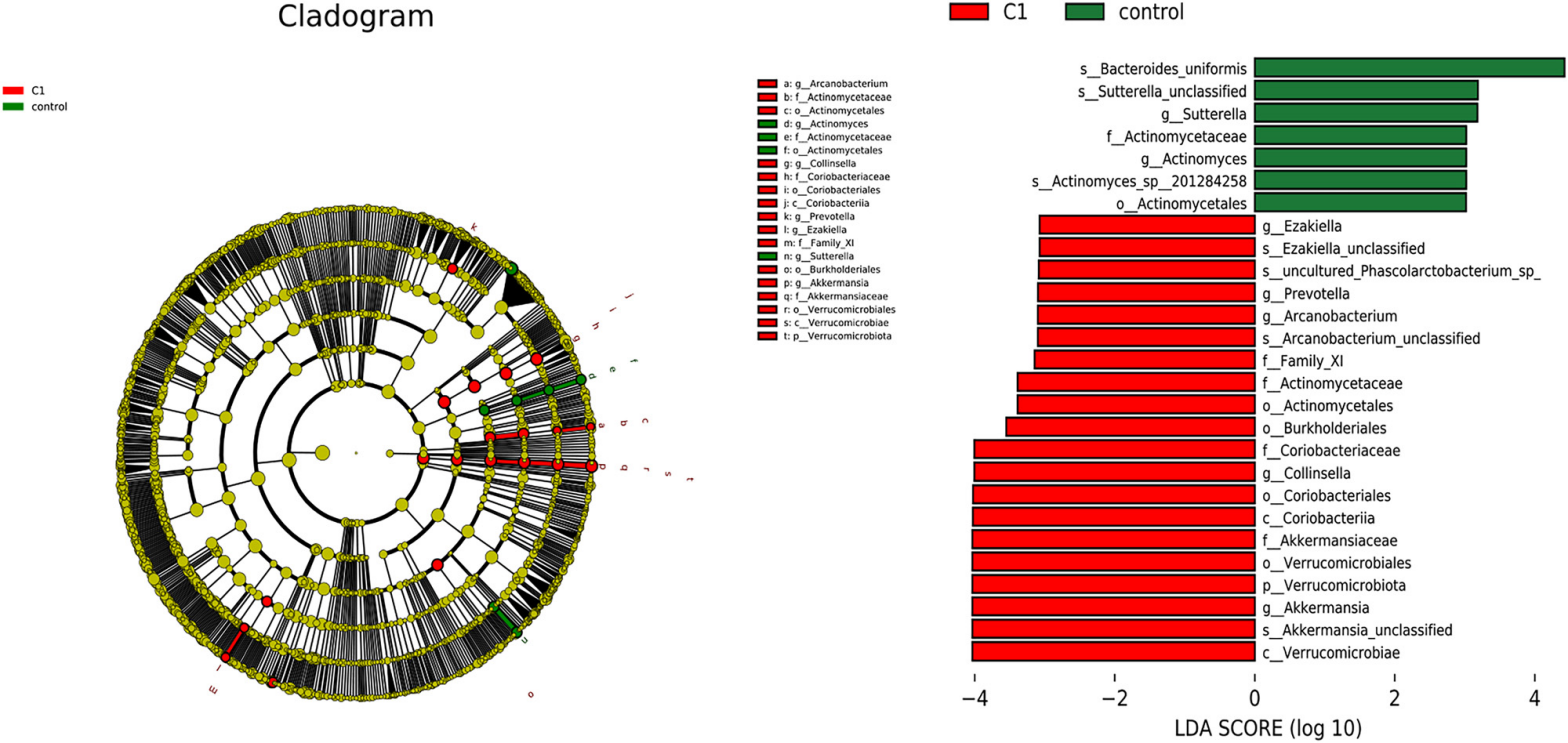
LEfSe analysis and histogram of LDA value distribution in pre-treatment primary hypothyroidism and healthy control group. LEfSe analysis mainly aims to compare the two groups and find species (biomarker) with significant difference in abundance between different groups. The species with an absolute value of LDA greater than 3 was defined as the keystone species, which was the species with significant differences in degree between different groups. The length of the bar graph indicates the influence degree of the species. C1: pre-treatment group; control: healthy control group.

**Figure 5:**
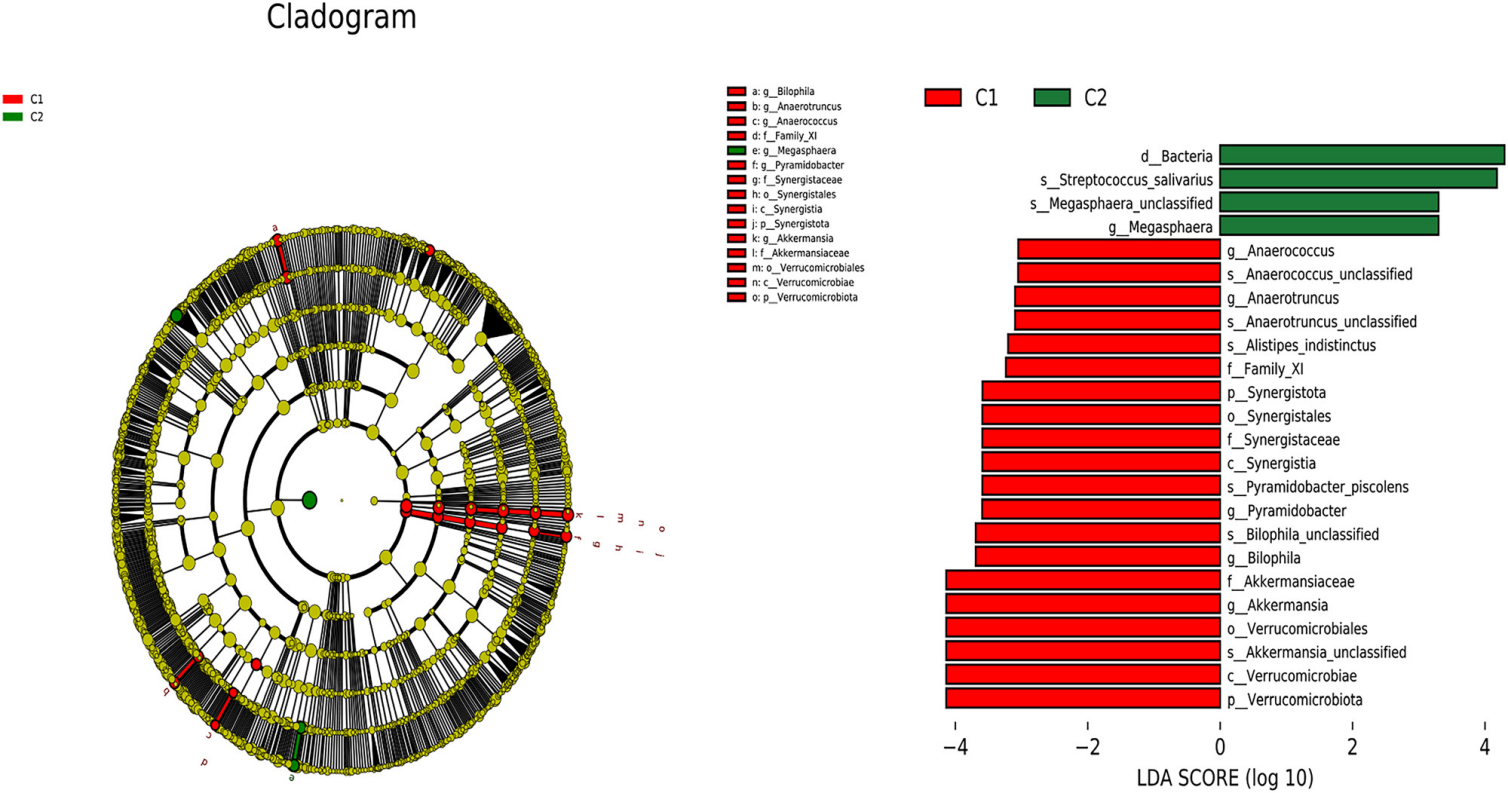
LEfSe analysis and histogram of LDA value distribution in pre-treatment and post-treatment primary hypothyroidism. LEfSe analysis mainly aims to compare the two groups and find species (biomarker) with significant difference in abundance between different groups. The species with an absolute value of LDA greater than 3 was defined as the keystone species, which was the species with significant differences in degree between different groups. The length of the bar graph indicates the influence degree of the species. C1: pre-treatment group; C2: post-treatment group.

Linear discriminant analysis effect size (LEfSe) was used to identify species with significant differences in abundance among the groups. Species with an LDA score greater than 3 were considered key species with significant differences among the groups. At the genus level, seven species differed significantly in relative abundance between the pre-treatment group and the healthy control group, with two genera enriched in the healthy control group (Sutterella and Actinomyces; *p* < 0.05) and five genera enriched in the pre-treatment group (Ezakiella, Prevotella, Anaerotruncus, Collinsella, and Akkermansia; *p* < 0.05), as shown in Figures 4. Six species exhibited significant differences in relative abundance between the pre-treatment and post-treatment groups, with five genera enriched in the pre-treatment group (Anaerococcus, Anaerotruncvs, Pyramidobacter, Bilophila, and Akkermansia; *p* < 0.05) and one genus enriched in the post-treatment group (Megasphaera; *p* < 0.05), as shown in Figure 5.

### 3.6 Correlation analysis between gut microbiota and clinical indicators

**Figure 6:**
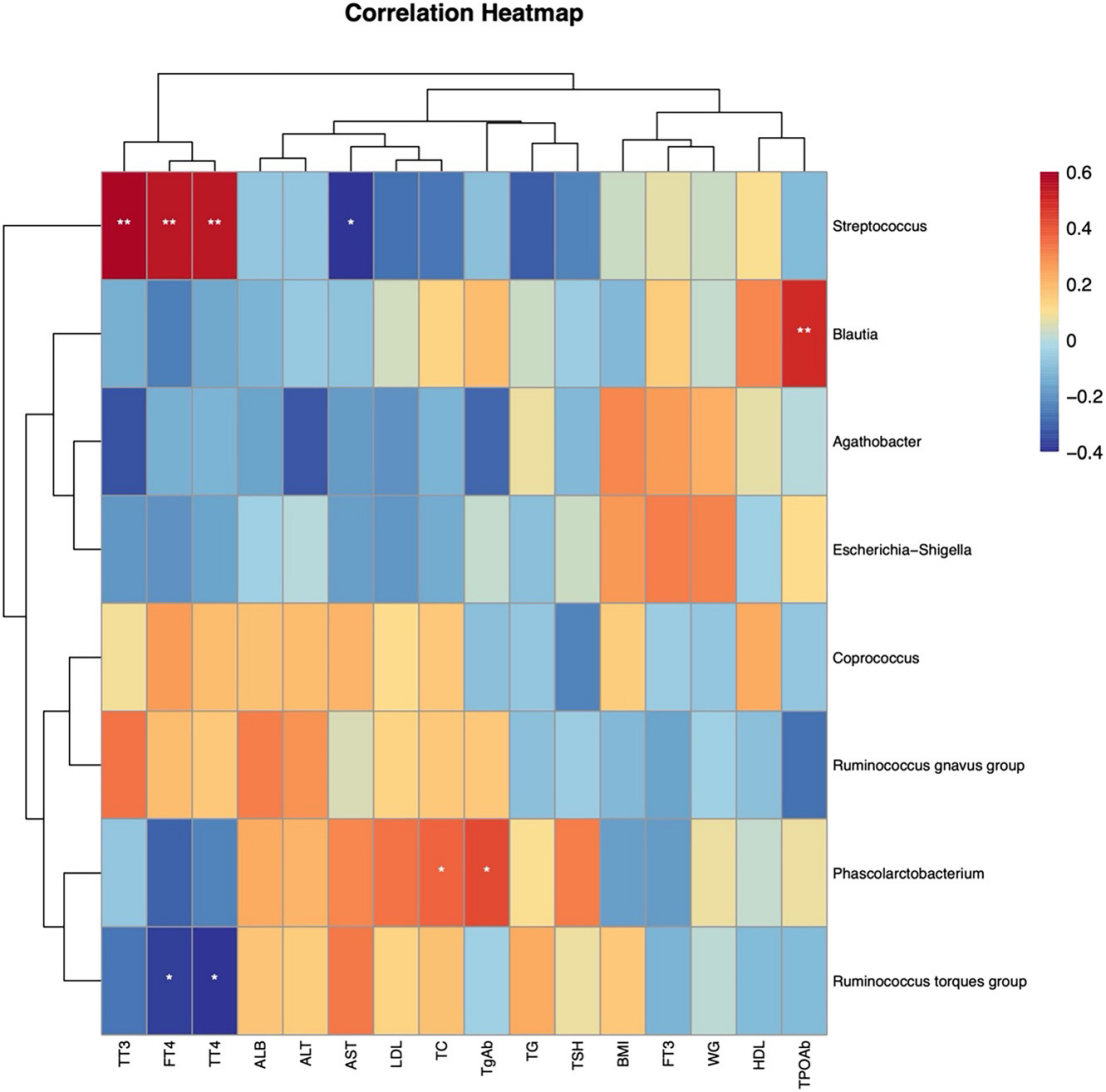
Heat map of Spearman correlation analysis between gut microbiota and clinical indicators in primary hypothyroidism before and after treatment. The heat map illustrates the intensity and direction of the correlation between the relative abundance of specific gut microbial (rows) and clinical parameters (columns) in patients with hypothyroidism. Warm color (red) indicates positive correlation, cool color (blue) indicates negative correlation, and white indicates no significant correlation. The intensity of the color reflects the correlation coefficient of size (*p <* *0.05, **p < 0.01).

Spearman correlation analysis was performed on the eight genera with *p* < 0.05 before and after LT-4 treatment and the clinical indicators with *p* < 0.05. Four genera were significantly correlated with clinical indicators. Streptococcus was positively correlated with TT3, TT4, and FT4 (*r* = 0.58, 0.54, 0.54, respectively; *p* < 0.05) and negatively correlated with AST (*r* = −0.44; *p* < 0.05). *R. torques* group was negatively correlated with TT4 and FT4 (*r* = −0.39, −0.37, respectively; *p* < 0.05). Phascolarctobacterium was positively correlated with TC and TgAb (*r* = 0.36, 0.43, respectively; *p* < 0.05). Blautia was positively correlated with TPOAb (*r* = 0.489; *p* < 0.05), as shown in Figure 6.

## 4 Discussion

The gut microbiota can influence gut barrier integrity, substance metabolism, and immune system function, playing a role in the pathogenesis and progression of autoimmune and metabolic diseases. Studies have shown that patients with Hashimoto’s thyroiditis exhibit increased thickness of microvilli in intestinal epithelial cells and greater spacing between adjacent microvilli compared to healthy controls, which is associated with increased intestinal permeability [7]. Thyroid hormones undergo deglucuronidation in the liver, enter the intestine via bile, and are broken down by β-glucuronidase produced by anaerobic bacteria in the gut. These hormones are then absorbed into the bloodstream and re-enter the liver via the portal vein, forming the enterohepatic circulation. Thus, the gut microbiota plays a crucial role in the enterohepatic circulation of thyroid hormones [8]. Hypothyroidism can cause mucinous edema in hepatocytes, leading to liver cell damage and abnormal liver enzyme levels. Thyroid hormones can regulate LDL receptor activity and affect lipid metabolism, resulting in elevated TC and LDL levels in hypothyroid patients. Our study indicates that after L-T4 treatment, levels of ALT, AST, TG, TC, and LDL significantly decreased in patients with primary hypothyroidism.

Our analysis of gut microbiota diversity in patients with Hashimoto’s thyroiditis and primary hypothyroidism shows significant statistical differences in the Chao1 index between the pre-treatment group and healthy controls, suggesting bacterial overgrowth in hypothyroid patients, consistent with Virili et al.’s findings [9]. After treatment, the Chao1 index decreased, indicating alleviation of bacterial overgrowth. Principal coordinate analysis (PCoA) of β-diversity revealed structural differences in microbiota composition between the healthy controls and pre- and post-treatment groups, highlighting significant differences in gut microbiota structure. Increased gut microbiota richness and diversity, reflected in small intestinal bacterial overgrowth (SIBO), has been reported to be significantly associated with hypothyroidism. Decreased gastrointestinal motility is a risk factor for SIBO development [10], and slow intestinal peristalsis in hypothyroid patients may alter substrate utilization and physicochemical conditions in the gut, potentially leading to dysbiosis [11].

Our study reveals that compared to healthy controls, patients with primary hypothyroidism have decreased abundance of Bacteroidota at the phylum level. Post-treatment, Bacteroidota abundance increased, while the abundances of Proteobacteria and Desulfobacteria decreased. Bacteroidota, one of the most abundant symbiotic phyla in healthy humans, includes commensals, probiotics, and pathogenic bacteria, and can produce acetate, promote mucin-related gene expression, enhance intestinal epithelial differentiation, and degrade polysaccharides for energy. A decrease in Bacteroidota can lead to a thinner mucosal layer and altered chemical barrier, resulting in abnormal gut function. In healthy mammals, the Firmicutes/Bacteroidetes (F/B) ratio remains relatively stable, so changes in the F/B ratio usually indicate a disturbance in homeostasis [12]. At the genus level, results show that newly diagnosed primary hypothyroid patients have lower abundances of *Bacteroides*, *Faecalibacterium* compared to healthy controls, with increased abundances of *Phascolarctobacterium*, *Collinsella*, *Klebsiella* and *Parabacteroides*. After L-T4 supplementation, the abundances of *Streptococcus*, *R. gnavus* group, *Megamonas* and *Faecalibacterium* increased, while the abundances of *Escherichia-Shigella*, *Agathobacter*, *R. torques* group, *Phascolarctobacterium*, and *Blautia* decreased. Research shows that *Bacteroides* plays a role in immune dysregulation, toxin production, and metabolic syndrome and is fundamental to gut microbial ecological balance [13]. A reduction in *Bacteroides* may lead to inflammation and autoimmune disease. Studies have also shown a significant reduction in *Bacteroides* in hyperthyroid patients [14]. Faecalibacterium is one of the most important bacteria in the human gut microbiota and a major producer of butyrate, playing a critical anti-inflammatory role and protecting the digestive system from intestinal pathogens [15]. Therefore, the decreased abundance of Faecalibacterium may reduce anti-inflammatory effects and promote autoimmune disease. Faecalibacterium and Veillonella produce butyrate, which induces the differentiation of regulatory T cells (Tregs), thus exerting anti-inflammatory effects and protecting against inappropriate immune responses [16]. Bifidobacteria also produce short-chain fatty acids (SCFAs) [17], which contribute to maintaining the hypothalamic-pituitary-thyroid axis and gastrointestinal function. Streptococcus, a normal microbiota of the oral cavity, upper respiratory tract, gut, and female reproductive tract, may impact host health if its abundance decreases [18]. Current research on Streptococcus is focused on digestion, respiration, and the nervous system, with limited studies on its role in autoimmune thyroid disease [19]. Correlation analysis shows that Streptococcus is positively correlated with TT3, TT4, and FT4, and negatively correlated with AST, suggesting a potential link between Streptococcus and thyroid function. *R. gnavus* group, as an anaerobic bacterium, can induce dendritic cells to produce inflammatory cytokines, most commonly reported in inflammatory bowel disease [20].

The study found that the abundance of the Megamonas in the intestinal microbiota of patients with Hashimoto’s thyroiditis was decreased. The reduction of this type of bacteria was often accompanied by a decrease in the levels of SCFAs. SCFAs play a crucial role in maintaining the integrity of the intestinal barrier, inhibiting systemic inflammation, and regulating immune balance. Therefore, the decrease in the abundance of the Megamonas may weaken this protective immunoregulatory microenvironment, thereby potentially exacerbating the autoimmune attack against the thyroid. The recovery of the abundance of the Megamonas after treatment in this study also reflects that the intestinal microbiota disorder has been improved to a certain extent, and the immune-inflammatory state has been alleviated [21].

*R. gnavus* group also has β-glucuronidase activity [22], which may affect the enterohepatic circulation of thyroid hormones and thus thyroid hormone absorption. In this study, its abundance increased after L-T4 treatment. *R. torques* group, a mucin-degrading bacterium, can degrade intestinal mucus and damage the gut barrier, often found at higher proportions in individuals with intestinal inflammation [23]. Research on *R. torques* group in thyroid diseases is limited; in this study, its abundance was higher before treatment, and correlation analysis showed that *R. torques* group is negatively correlated with TT4 and FT4, potentially related to gut barrier damage affecting thyroid function.

LEfSe analysis identified Collinsella as a biomarker in primary hypothyroid patients before treatment, with levels significantly higher than in the control group. Studies show that Collinsella can increase intestinal mucosal permeability, regulate intestinal motility, reduce the expression of tight junction proteins in epithelial cells, and release pro-inflammatory cytokines such as IL-17, potentially leading to dysbiosis and immune system activation [24], which may contribute to the development of autoimmune diseases, including Hashimoto’s thyroiditis with primary hypothyroidism. LEfSe analysis also identified Akkermansia as a biomarker in primary hypothyroid patients both before and after treatment. Akkermansia is considered a potential probiotic for the gastrointestinal tract and is closer to the intestinal epithelial cells compared to other gut microbes, making its metabolic products such as propionate more readily absorbed by the host. Akkermansia is closely linked to host metabolic function and immune response and enhances the integrity and thickness of the mucosal layer, promoting gut health. Therefore, a low abundance of Akkermansia can lead to thinner mucus and weakened microbial barrier function, making it easier for toxins to penetrate the host and lead to disease [25].

A study involving 60 primary hypothyroid patients treated for 8 weeks showed that patients using L-T4 alone required higher doses of L-T4 compared to those using L-T4 in combination with probiotics [26]. Spaggiari et al. found that lactobacilli and bifidobacteria significantly reduced the L-T4 requirements in hypothyroid patients and helped decrease serum thyroid hormone fluctuations [27]. These studies suggest that combining L-T4 with probiotics can reduce the dosage of L-T4 needed for hypothyroid patients, further demonstrating the impact of gut microbiota on thyroid function.

## 5 Conclusions

This study further elucidates changes in gut microbiota and related clinical indicators before and after treatment in primary hypothyroidism patients, indicating that dysbiosis is closely related to the development of Hashimoto’s thyroiditis with hypothyroidism and provides evidence for the thyroid-gut axis theory. The limitations of this study include: (1) The small sample size may introduce bias, limiting the generalizability and conclusiveness of the results; (2) Sample collection was limited to the Kunming area, without considering regional, seasonal, or dietary influences.
